# The structural changes of pharyngeal airway contributing to snoring after orthognathic surgery in skeletal class III patients

**DOI:** 10.1186/s40902-017-0120-6

**Published:** 2017-08-05

**Authors:** Jung-Eun Park, Seon-Hye Bae, Young-Jun Choi, Won-Cheul Choi, Hye-Won Kim, Ui-Lyong Lee

**Affiliations:** 1 Department of Orthodontics, Dental Center, Chung-Ang University Hospital, Seoul, South Korea; 2 Department of orthodontics, Estar dental clinic, Seoul, Republic of Korea; 3 Department of Oral and Maxillofacial Surgery, Dental Center, Chung-Ang University Hospital, 224-1 Heukseok-dong, Dongjak-gu, Seoul, South Korea

**Keywords:** Bimaxillary surgery, Class III malocclusion, Obstructive sleep apnea, Posterior airway space, Snoring

## Abstract

**Background:**

Two-jaw surgery including mandibular and maxillary backward movement procedures are commonly performed to correct class III malocclusion. Bimaxillary surgery can reposition the maxillofacial bone together with soft tissue, such as the soft palate and the tongue base. We analyzed changes of pharyngeal airway narrowing to ascertain clinical correlations with the prevalence of snoring after two-jaw surgery.

**Methods:**

A prospective clinical study was designed including a survey on snoring and three-dimensional (3D) computed tomography (CT) in class III malocclusion subjects before and after bimaxillary surgery. We conducted an analysis on changes of the posterior pharyngeal space find out clinical correlations with the prevalence of snoring.

**Results:**

Among 67 subjects, 12 subjects complained about snoring 5 weeks after the surgical correction, and examining the 12 subjects after 6 months, 6 patients complained about the snoring. The current findings demonstrated the attenuation of the largest transverse width (LTW), anteroposterior length (APL), and cross-sectional area (CSA) following bimaxillary surgery given to class III malocclusion patients, particularly at the retropalatal level. The average distance of maxillary posterior movements were measured to be relatively higher (horizontal distance 3.9 mm, vertical distance 2.6 mm) in case of new snorers.

**Conclusions:**

This study found that bimaxillary surgery could lead to the narrowing of upper airway at the retropalatal or retroglossal level as well as triggering snoring in subjects with class III malocclusion. Based on the current clinical findings, we also found that upper airway narrowing at retropalatal level may contribute to increasing the probability of snoring and that polysonography may need to be performed before orthognathic surgery in subjects with class III malocclusion.

## Background

Snoring is the resulting sound caused by the structure of the upper airway during sleep. It typically happens on inspiration but may also occur on expiration. Habitual snoring is found to be common since it occurs in 44% of males and 28% of females between the ages of 30 and 60 years among the general population [1]. Occasional snoring is an almost universal symptom [2]. Snoring is caused by increased upper airway resistance. It can be regarded as a sign of obstructive sleep apnea (OSA), a sleep disorder involving obstructive apneas and hypopneas that occurs when there is a sufficient upper airway resistance which may disrupt sleep [3].

Snoring may also be linked with conditions which narrow the upper airway, such as obesity, nasal congestion, craniofacial abnormalities, hypothyroidism, acromegaly, and adenotonsillar hypertrophy. These conditions may result in snoring without OSA or snoring that is presented as a symptom of OSA [1].

Class III malocclusions may involve skeletal discrepancies including a prognathic mandible with or without a maxillary protrusion and class III malocclusion patients account for a great proportion of those who are in need of surgical-orthodontic treatment because of esthetic and functional problems [4]. Mandibular setback osteotomy has been routinely conducted as an orthognathic surgical procedure for the treatment of mandibular prognathism and bimaxillary orthognathic surgery including mandibular setback, and maxillary backward movement procedures are typically used to treat class III malocclusion [5]. For patients presenting with skeletal class III malocclusion, bimaxillary surgery can correct the position of the orofacial skeletal as well as soft tissue components including the soft palate and the tongue base.

According to previous studies conducted by some authors, bimaxillary surgery [6, 7] is expected to narrow the posterior airway space (PAS). The two-jaw surgery accompanied by maxillary posterior differential impaction can decrease the total volume of airway in the patients presenting with skeletal class III [8]. As a result, patients who undergo bimaxillary surgery are likely to be suffering from sleep-disordered breathing particularly, which is caused by the narrowing of the PAS and airway collapse while sleeping [9–11].

Nevertheless, the potential effect of PAS narrowing induced by bimaxillary surgery in the advancement of sleep-disordered breathing or OSA is a subject still under debate, along with other contentious questions such as whether changes are caused in sleep architectures by bimaxillary surgery.

Few functional assessments have been conducted as to the prevalence rate of OSA using pre- and post-operative sleep studies in patients presenting with dentofacial deformities. In particular, the mutual clinical relation between PAS narrowing after surgery and development of snoring or sleep apnea in class III malocclusion subjects has not been clearly investigated.

Accordingly, in this article, a prospective clinical study was designed including a survey on snoring and three-dimensional (3D) computed tomography (CT) in class III malocclusion subjects before and after bimaxillary surgery. We conducted an analysis on alterations of the PAS to find out clinical correlations with the prevalence of snoring.

## Methods

### Study subjects and surgical technique for treatment of maloccusion

Sixty-seven adult subjects who received bimaxillary surgery from March 2013 to June 2013 voluntarily took part in the study. A survey on snoring and volumetric measurement was conducted prospectively. Written informed consent was gained from each participant, and the study was in accordance with the Declaration of Helsinki. This study was approved by the institutional review board (C2015022).

We designed a prospective study to recruit subjects given a diagnosis of skeletal class III malocclusions and scheduled to receive bimaxillary surgeries at the department of oral and maxillofacial surgery. For the primary treatment, all of the enrolled subjects underwent a Le Fort I osteotomies and the L-plates were fixed to the pyriform aperture and the zygomatic buttresses bilaterally. Next, sagittal split ramus osteotomies were performed. Each titanium plate had three holes, and the semi-rigid fixation was done between split segments using titanium screws. Subjects, who received bimaxillary surgery, showed a body mass index above 30 kg/m^2^, or already knew about their OSA or snoring in advance of surgery were excluded. Moreover, subjects presenting with severe septal deviation, chronic hypertrophic rhinitis, and tonsil hypertrophy that could have influence on the prevalence of snoring or OSA were excluded in the present study.

Patients with severe structural facial asymmetry where the menton deviation is more than 4 mm, patients who have congenital structural defect such as acromegaly or cleft lip or palate, and patients who are under 18 years old are also excluded from the study. Also, patients who had their maxilla advanced after Le Fort I osteotomy were excluded from the study as well.

### Criteria of existence/absence of snoring

Patients who responded “strongly agree” and “agree” among the four choices, “strongly agree,” “agree,” “disagree,” and “strongly disagree,” to the question asking whether the patient snores (more) after the surgery on a written survey 5 weeks post-op and 6 months post-op, were deemed to have developed snoring or worsened of the existing condition. Among such patients, patients who have improved after 6 months were excluded.

### CBCT for the measurement of airway and skeletal structure

All patients received cone-beam computed tomograms (CBCT, 3D eXam, Kavo Dental GmbH, Biberach, Germany) in advance of surgery (T0) and 6 months after surgery (T1). CBCT images were taken with each patient in an upright posture when Frankfort horizontal plane was parallel to the floor, in a centric occlusion [11]. Using 0.4 mm voxel size and 512 × 512 matrices, the CBCT data (detector field of view (FOV) of 12-inch, 120 kVp, 11 mAs, 17.8 s scan time) of the maxillofacial regions were acquired. Patient data were exported in DICOM (Digital Imaging and Communications in Medicine) format, and reconstruction of images into 3D using software was done (Invivo5, Anatomage, San Jose, CA, USA).

**Fig. 1:**
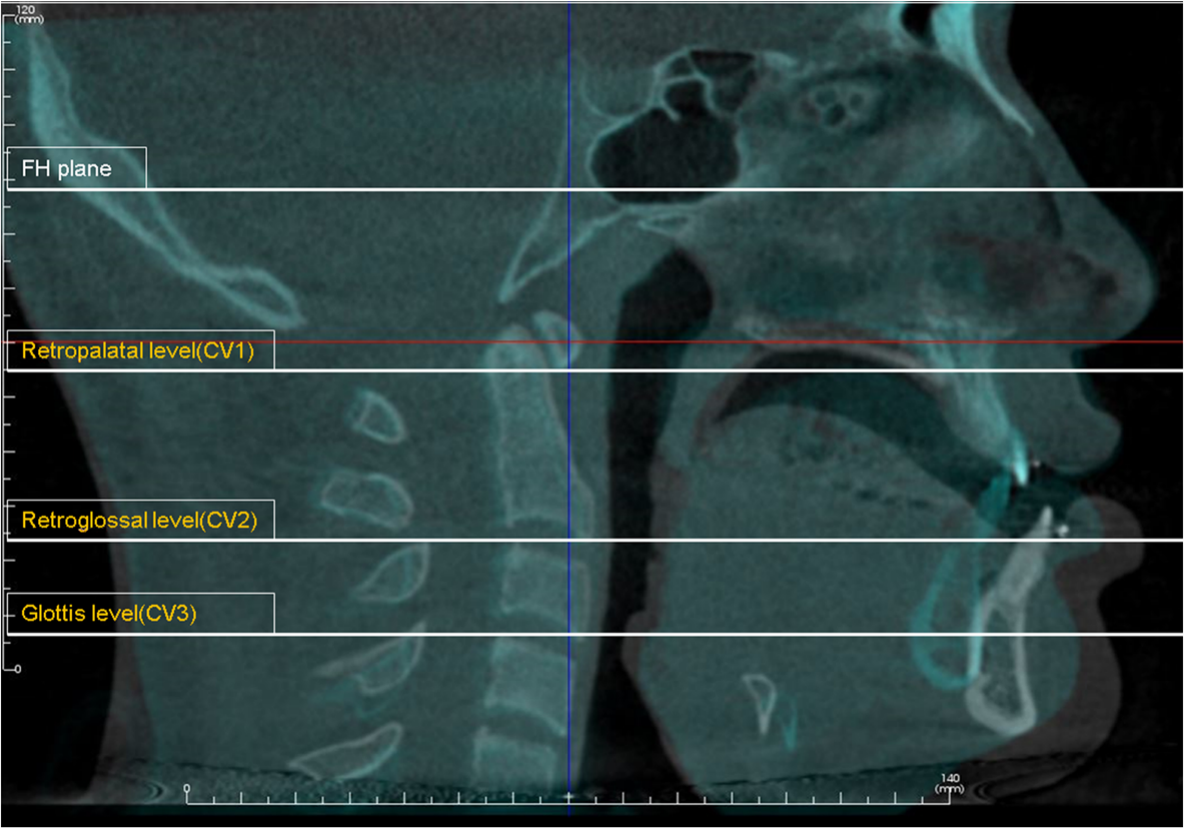
Reference planes. CV1, CV2, and CV3 planes were parallel to the Frankfort horizontal plane

**Fig. 2:**
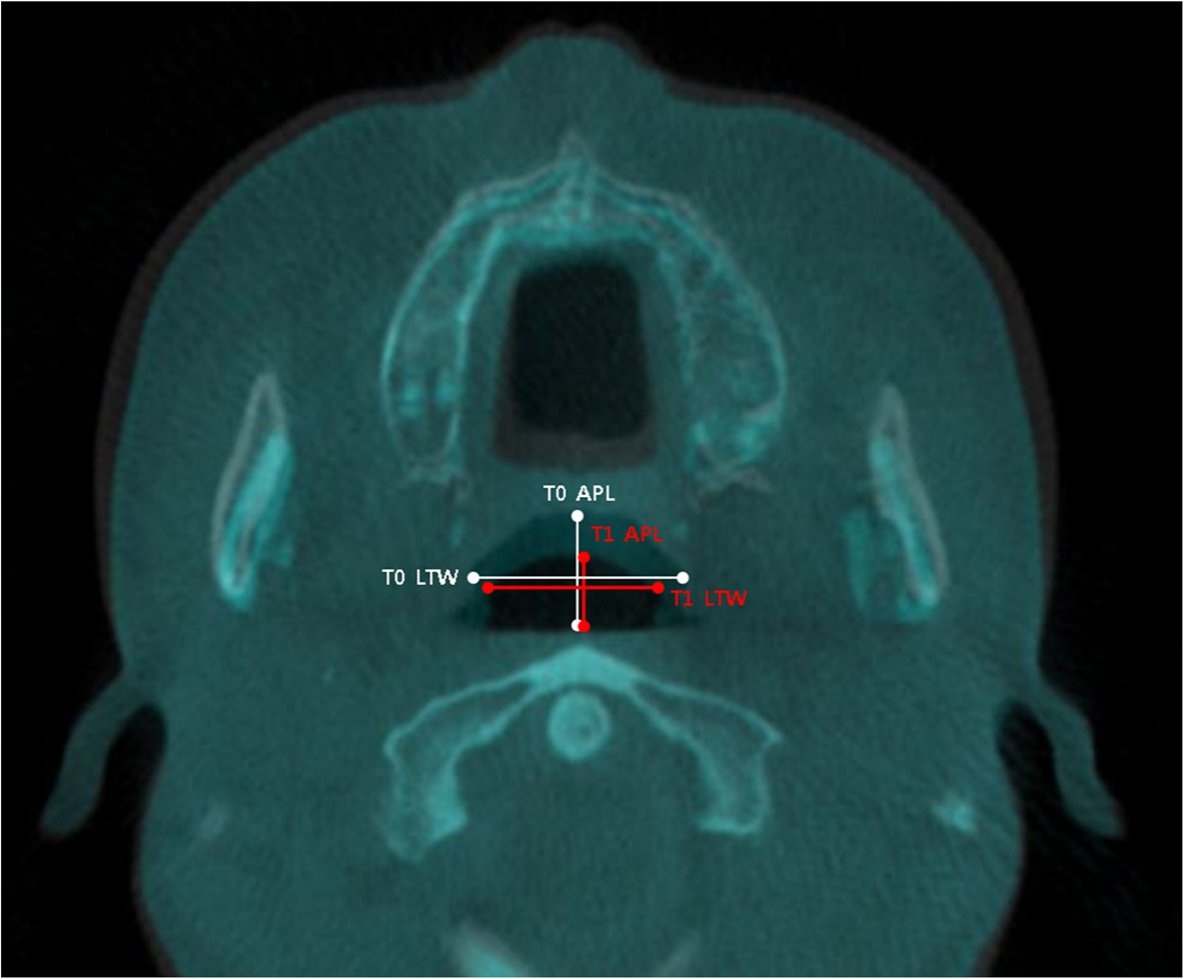
Evaluation of the pharyngeal airway changes. The largest transverse width (*LTW*) and anteroposterior length (*APL*) on each axial plane (CV1, CV2, CV3) were measured at T0 and T1

**Fig. 3:**
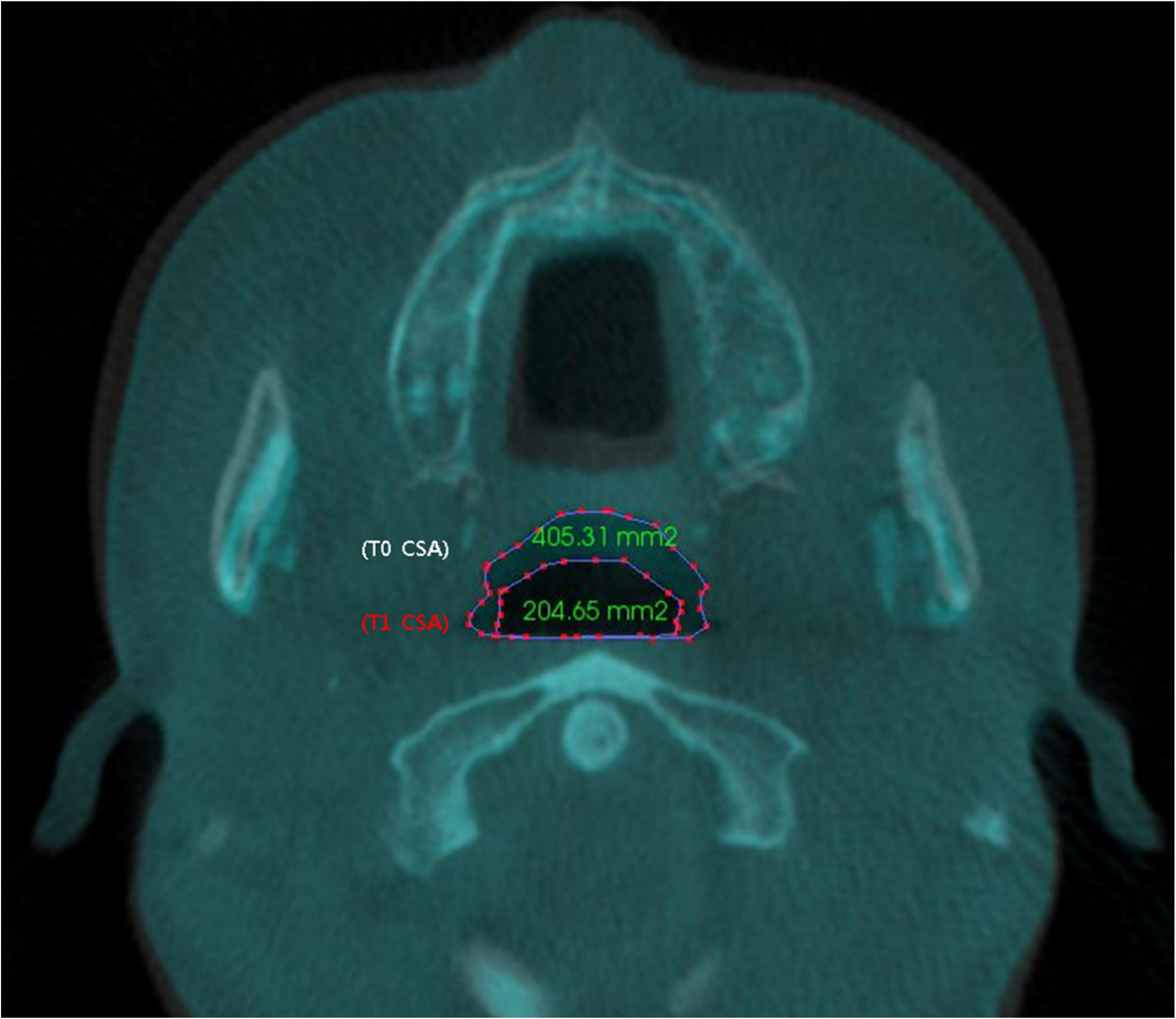
Evaluation of the pharyngeal airway changes. Cross-sectional area (*CSA*) on each axial plane (CV1, CV2, CV3) were measured at T0 and T1

To conduct evaluation of changes in the pharyngeal airway, the measurement of the largest transverse width (LTW), anteroposterior length (APL), and cross-sectional area (CSA) on each axial plane (CV1, CV2, CV3) at T0 and T1 (Figs. 1, 2, and 3) was performed. In measuring of changes in the LTW, APL, and CSA, a method described in this study with the superimposition module of the Invivo 5 program (9) was used. Then, it was followed by a superimposition of pre- and post-operative CBCT data by point registration and automatic voxel-by-voxel registration at the craniomaxillofacial area unchanged by orthognathic surgery. On the images that were superimposed, LTW, APL, and CSA in the axial CV1, CV2, and CV3 were measured.

**Fig. 4:**
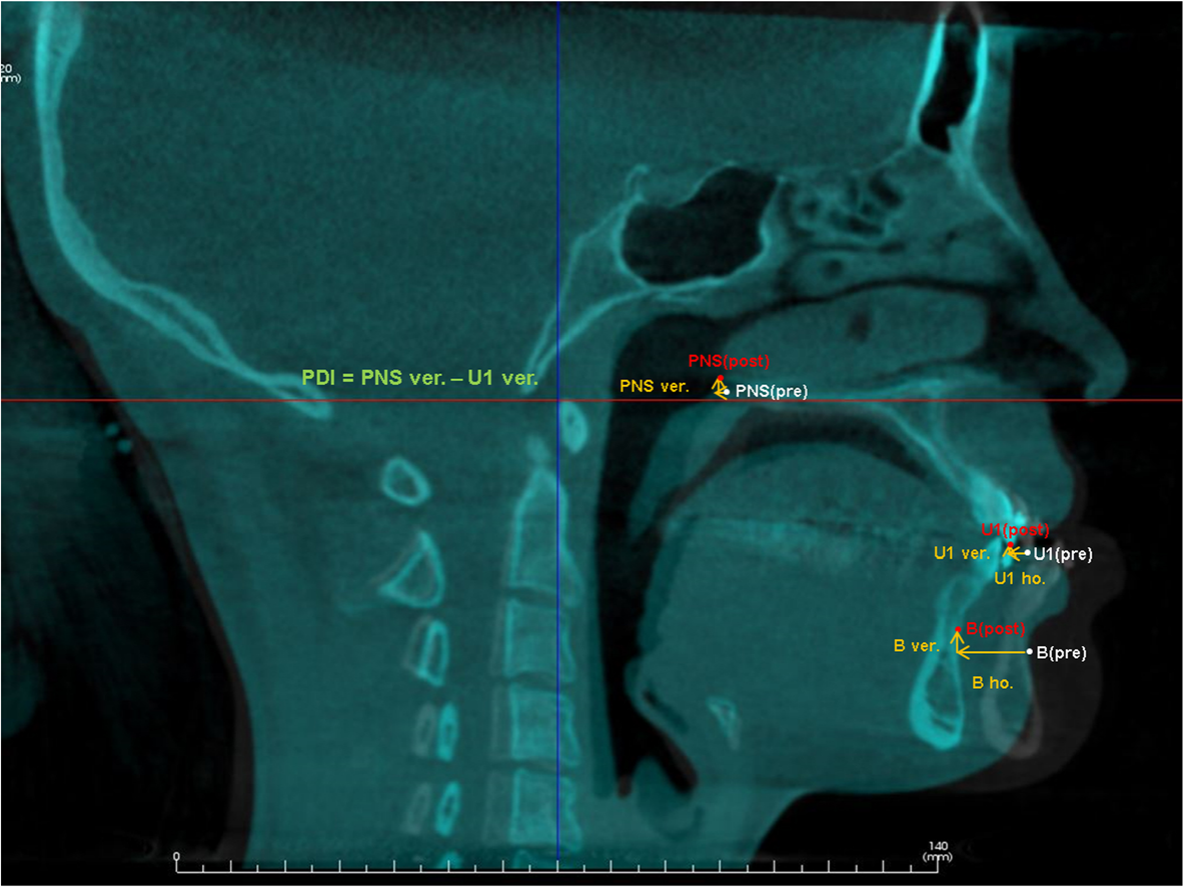
Landmark measurements for surgical skeletal movements. The distances of maxillary and mandibular movements between prior to and after two-jaw surgery were measured at PNS, U1, and B point. *U1* maxillary first molar root apices, *B points* innermost curvature from chin to alveolar bone junction, *PNS* posterior nasal spine, *PDI* posterior differential impaction)

The evaluation of skeletal changes following bimaxillary surgery was conducted based on the same superimposition and coordinate system which was used to measure airway change. To evaluate movement of the maxilla and mandible, the calculation of coordinate values of the U1 (incisal edge of right upper central incisor), B (B point), PNS (posterior nasal spine) was made from the midsagittal view (Fig. 4) and posterior or superior movements were assigned positive values. B point is considered to be the point at the most concave location on mandibular symphysis and posterior differential impaction indicates the degree of maxillary clockwise rotation.

### Statistical analysis

The APL and LTW were measured before and after surgery at levels CV1, CV2, and CV3, and the average and standard deviation of the difference of APL and LTW before and after surgery were calculated. In order to find the cause that affects the snoring the most, multiple logistic regression was used (MATLAB 2013 program). APL and LTW after surgery, the amount and rate of change in APL and LTW before and after surgery, and multiplied value or the amount/rate of change were set to be explanatory variable, and snoring was set to be the explained variable. Using multiple logistic regression, the regression analysis was performed at 1 and 5% significance level to find out how the explanatory variable contributes to the occurrence rate of a specific event (explained variable). Moreover, to investigate the relationship among the factors that are set to be explanatory variables, Pearson correlation coefficient was used.

## Results

### Clinical characteristics of the studied subjects presenting with class III malocclusion

The present study conducted an investigation on 67 subjects presenting with class III malocclusion including 24 men and 43 women. For primary treatment for class III malocclusion, all the subjects underwent Le Fort I osteotomy and sagital split ramus osteotomy of the mandible. The average age of the subjects was 24.3 years, and mean body mass index was 22.1 kg/m^2^ (Table 1). There were no subjects who were given a diagnosis of sleep breathing disorders including OSA and primary snoring in advance of surgery.

**Table 1:** Patient characteristics

	Snoring group	Non-snoring group	*p* value
	(*n* = 6)	(*n* = 61)	
Age	24.30 ± 1.30	24.50 ± 2.21	0.790
Gender (M:F)	4:2	20:41	0.255
Height	169.32 ± 5.30	167.32 ± 11.03	0.672
Weight	61.21 ± 2.76	57.51 ± 5.65	0.125
BMI	22.11 ± 1.32	22.74 ± 2.72	0.717

Data are presented as mean ± standard deviation

### Measurement of the anterior-posterior length (APL), lateral transverse width (LTW), and cross-sectional area (CSA) of the upper airway

After surgery, the APL of the airway decreased by 2.3 and 1.6 mm in CV1 and CV2, respectively, and increased by 0.34 mm in CV3. The LTW of airway decreased by 1.9, 2.3, and 0.7 mm in CV1, CV2, and CV3, respectively. Except the APL in CV3, all other APL and LTW measurements had a tendency to decrease, and the greatest changes were observed in APL of CV1 and LTW of CV2. The CSA of airway decreased by 95.2 mm^2^ in CV1, 68.2 mm^2^ in CV2, and 9.5 mm^2^ in CV3 (Table 2).

**Table 2:** Changes in pharyngeal configuration and CSA

		T 0		T 1		T1–T0	
		Average	SD	Average	SD	Average	SD
C1 (retropalatal)	LTW	29.9	5.9	27.7	5.8	−2.3	3.9
	APL	15.5	3.7	13.6	3.9	−1.9	2.1
	CSA	389.5	4.3	294.3	5.6	−95.2	3.2
C2 (retroglossal)	LTW	27.1	5.7	25.5	5.6	−1.6	3.3
	APL	13.5	4.1	11.2	3.4	−2.3	3.3
	CSA	278.9	4.8	210.7	4.6	−68.2	2.4
C3 (glottis)	LTW	30.9	4.7	31.3	4.9	0.3	2.5
	APL	11.4	2.9	10.7	3.9	−0.7	2.8
	CSA	233.4	2.1	223.9	4.5	−9.5	2.4

*APL* anterior-posterior length, *LTW* lateral transverse width, *CSA* cross-sectional area

The current findings demonstrated the attenuation of the APL, LTW, and CSA following bimaxillary surgery given to class III malocclusion patients, particularly at the retropalatal and retroglossal level.

### Snoring after surgical correction of class III malocclusion

Among 67 subjects, 12 subjects complained about snoring 5 weeks after the surgical correction, and examining the 12 subjects after 6 months, 6 patients complained about the snoring. There was no patient that complained about relapse of snoring after 6 months of surgical correction if they have indicated that they had no snoring 5 weeks after surgical correction.

### Analysis of structural changes of pharyngeal airway contributing to snoring

The absolute value of APL and LTW after surgical correction was revealed to have no significant relationship in regards to snoring, and CSA value after surgery showed 5% significance with manifestation of snoring in CV1 (Table 3).

**Table 3:** Correlation between post-operative airway configuration and snoring

	T1	Beta	SD	*p* value
C1 (retropalatal)	LTW	−0.445	0.2338	0.0570*
	APL	0.1079	0.217	0.6192
	CSA	−0.122	0.231	0.0432**
C2 (retroglossal)	LTW	0.2455	0.2336	0.2933
	APL	−0.8229	0.4745	0.0829*
	CSA	−0.432	0.344	0.3234
C3 (glottis)	LTW	−0.0969	0.1607	0.5468
	APL	0.2811	0.4153	0.4986
	CSA	−0.121	0.3443	0.1219

***,**,* represent 1, 5, 10% significance level, respectively

*APL* anterior-posterior length, *LTW* lateral transverse width, *CSA* cross-sectional area

The change of APL and LTW before and after surgical correction was revealed to have no significant relationship in regards to snoring, and the change of CSA before and after surgery showed 5% significance with manifestation of snoring in CV1 (Table 4).

**Table 4:** Correlation between changes of airway configuration and snoring

	T1–T0	Beta	SD	*p* value
C1 (retropalatal)	LTW	−1.49	0.7738	0.0542*
	APL	−1.702	0.8928	0.0566*
	CSA	−0.0294	0.0121	0.0149**
C2 (retroglossal)	LTW	−0.1085	0.4528	0.8107
	APL	−0.0631	0.3974	0.8738
	CSA	−0.0018	0.005	0.7183
C3 (glottis)	LTW	0.2066	0.4099	0.6143
	APL	−0.5368	0.4605	0.2437
	CSA	−0.001	0.0061	0.8682

***,**,* represent 1, 5, 10% significance level, respectively

As for the ratio of change before and after the surgery in regards to measurements before surgery, the ratio of change in APL and CSA in CV1 showed significance in occurrence of snoring at 1% significance level (Table 5).

**Table 5:** The correlation of the proportion of difference between before and after surgery to the values before surgery

	T1–T0/T1	Beta	SD	*P*-value
C1 (retropalatal)	LTW	−0.7602	0.2819	0.0070 ***
	APL	−0.1464	0.084	0.0813
	CSA	−0.0294	0.0121	0.0149 **
C2 (retroglossal)	LTW	−0.0066	0.1285	0.9593
	APL	0.0222	0.0715	0.7557
	CSA	−0.0018	0.005	0.7183
C3 (glottis)	LTW	0.1733	0.1382	0.2098
	APL	−0.1424	0.0729	0.0507 *
	CSA	−0.001	0.0061	0.8682

***,**,* represent 1, 5, 10% significance level, respectively

For the assessment of skeletal movements of maxilla and mandible according to bimaxillary surgery in class III malocclusion, we made a calculation on the horizontal and vertical distances of maxilla (U1) and mandible (B) from each landmark. We also measured the degree of maxillary clockwise rotation at PNS through PDI values. Interestingly, the average skeletal movements of horizontal and vertical distances were measured to be 1.8 and 2.2 mm and PDI value was 3.2 mm in class III malocclusion subjects without showing advancement of snoring. However, the average distance of maxillary posterior movements was measured to be relatively higher (horizontal distance 3.9 mm, vertical distance 2.6 mm) in case of new snorers (Table 6).

**Table 6:** The average skeletal movements of subjects after bimaxillary surgery

Skeletal movement	Snoring group	Non-snoring group	*p* value
U1 set back (mm)	3.9 ± 1.53	1.8 ± 1.87	0.0121**
U1 up (mm)	2.6 ± 1.93	2.2 ± 1.45	0.665
PDI (mm)	3.8 ± 1.57	3.2 ± 1.05	0.412
B set back (mm)	9.7 ± 3.59	10.3 ± 4.33	0.772
B up (mm)	6.2 ± 2.64	7.6 ± 3.54	0.296

Landmark measurements for surgical skeletal movements. The distances of maxillary and mandibular movements between prior to and after bimaxillary surgery were measured at PNS, U1, and B point.

*U1* maxillary first incisor insical edge, *B points* innermost curvature from chin to alveolar bone junction, *PNS* posterior nasal spine, *PDI* posterior differential impaction

***,**,* represent 1, 5, 10% significance level, respectively

In order to see whether each variable has correlation to each other, an analysis using Pearson correlation coefficient was done, and it was revealed that the difference of APL and LTW before and after surgery at each level is not dependent on one another; these were independent variables (Table 7).

**Table 7:** Pearson correlation coefficient indicating the correlation between each factor

(T1–T0)		CV1		CV2		CV3	
		ΔAPL	ΔLTW	ΔAPL	ΔLTW	ΔAPL	ΔLTW
CV1	ΔAPL	1	0.4228	0.3363	0.2944	0.2642	0.0365
	ΔLTW		1	0.1658	0.2732	−0.0084	0.0649
CV2	ΔAPL			1	0.4621	0.3637	0.1642
	ΔLTW				1	0.1583	0.3508
CV3	ΔAPL					1	0.3222
	ΔLTW						1

***,**,* represent 1, 5, 10% significance level, respectively

## Discussion

This prospective study found that bimaxillary surgery could lead to the narrowing of upper airway at the retropalatal or retroglossal level as well as triggering snoring in subjects with class III malocclusion.

Based on the current clinical findings, we also found that upper airway narrowing at retropalatal level may contribute to increasing the probability of snoring and airway configuration and that sleep study may need to be conducted before orthognathic surgery in subjects with class III malocclusion.

The exact cause of OSA is unknown, yet it seems to be originated from multifactorial origin. Possible etiologies include neurologic control of upper airway, pharyngeal structures [12], and obesity [13], and possibly others [14, 15]. Though the cause of OSA is still unknown, it is known that pharyngeal airway volume decreases while airway resistances increase in apneic patients compared to normal population [13].

There were many previous studies on mandicular setback surgery and airway changes in skeletal class III malocclusion patients. Tselnik and Pogrel reported that airway narrows at oropharyngeal level when mandibular setback surgery is performed [16]. Liukkonen et al. also reported that airway size decreased at oropharyngeal and hypopharyngeal levels when mandibular setback surgery was performed [17]. Though there are not many reports of clinical respiratory disturbance caused by airway size decrease, Riley and Powell reported when patients with mandibular prognatism receives maxillary retrusion surgery, there is a possibility of developing OSA due to the airway size decrease [18]. On the other hand, Wenzel et al. reported that pharyngeal airway does not necessarily increase the airway resistance [19, 20].

Upper airway narrowing following orthognathic surgeries has recently been attracting attention from orthognathic researches, focused on patients who undergo bimaxillary surgery and can develop sleep-breathing disorders including OSA according to structural alterations of bone, muscle, and soft tissue around the pharynx.

Other studies prior to the study measured the changes of anteroposterior of airway using two-dimensional images obtained from cephalogram. However, a setback for such two-dimensional evaluations is that it can be affected by the repositioning of maxillofacial structures. Park et al. showed that linear analysis showed the decrease of pharyngeal depth and airway space when mandibular setback surgery is performed, but there were no significant decrease in linear, area, or volumetric measurements of the nasopharyngeal or oropharyngeal airway when volumetric analysis was performed [21].

It was noted that such results may have been caused by a physiological deformation, which is caused by the effort of maintaining airway volume upon the sagittal compression. Since there is a clear limitation on the two-dimensional measurement, this study is thought to have accurately analyzed the change in airway after surgery by measuring the airway change using superimposition of 3D video through CBCT.

The result of this study shows that if CSA of CV1 decreases after a bimaxillary surgery, snoring significantly increases. Such decrease of APL and LTW in CV1 is thought to be caused by the retrusion of soft palate caused by retrusion of maxilla. The decrease of APL and LTW in CV2 and CV3, when compared with the influence of decrease of APL and LTW in CV1, does not influence on the snoring as much. In other words, the retrusion of tongue and epiglottis does not have much effect on snoring compared to the retrusion of soft palate.

In most cases of orthognathic surgery of skeletal class III malocclusion, the surgery aims mandibular retrusion by causing maxillary retrusion and posterosuperior rotation. In conclusion, it is possible that the involvement of maxillary retrusion when performing a corrective orthognathic surgery for mandibular retrusion would cause retrusion of soft palate, and thus, causing snoring. Therefore, it is crucial to screen patients with airway APL at the level of soft palate, have narrow LTW, or patients who already snores before performing the surgery. If a retrusion, which does not involve posterosuperior rotation of maxilla in STO, is planned, it would be necessary to sufficiently inform the patient of the possibility of snoring. Moreover, it would be necessary to reconsider anterior segmental osteotomy to decrease the shift of maxilla.

This research has many limitations, as it is difficult to measure continuous, actual respiratory functional change by conducting a survey on snoring. Also, this research evaluated the patients up to 6 months after the surgery, but there was no evaluation of airway change that can happen due to the physiological adaptation 6 months after surgery. Therefore, there should be an addition research on analyzing the dynamics of actual respiratory process as well as the physiological adaptation of the airway after the surgery.

As some of the researches performed to standardize the normal value for the pharyngeal space, Samman et al. and Hochban et al. reported that since skeletal class III malocclusion patients have wider upper airway compared to the normal population, the decrease caused by surgery would still put them in normal range [22–24]. Therefore, the occurrence of OSA is rare after corrective surgery of skeletal class III malocclusion patients, and if such clinical problems as OSA occurred, then the sudden decrease of airway volume would not be the cause, while it is probable that other factors of the patient would have caused such symptoms. Therefore, if any clinical symptoms persist, objective examinations of respiratory resistance while sleeping, such as polysomnography, would be needed, and subjective tests including survey would be necessary [25–28].

The change in dentofacial morphology is reflected closely in the reduction of upper airway induced by surgery and a decrease in the upper airway can cause changes in sleep architectures of subjects with class III malocclusion.

In particular, the reduced dimension at the retropalatal level was found to be more extensive in subjects who showed advanced symptom of snoring after surgery. Furthermore, there were larger horizontal movements of maxilla observed in subjects with class III malocclusion who developed snoring following orthognathic surgery. The present study showed that the pharyngeal airway was narrowed by the posterior movement of the maxilla. A greater magnitude of maxilla or mandibular backward positioning may also have influenced six of the studied subjects presenting with postoperative snoring. Therefore, in advance of surgical treatment to correct class III malocclusion for minimizing risk of postoperative sleep-breathing disorders, the careful evaluation on the cephalometric analysis should be conducted and a risk of excessive setback of the maxilla and mandible which may elevate the probability of sleep breathing disorders is high.

## Conclusions

Bimaxillary surgery induced postoperative narrowing of the upper pharyngeal airway and leaded to snoring after surgery in some subjects with class III malocclusion. The airway configuration and surgical planning should be exquisitely conducted based on the upper airway for prevention of sleep-related disorders in subjects with class III malocclusion, and a plan must be established to enable the forward repositioning of the maxilla during the surgery conducted through anterior segmental osteotomy or orthodontic premolar extraction in collaboration with the orthodontist.
