# Correction to: The structural changes of pharyngeal airway contributing to snoring after orthognathic surgery in skeletal class III patients

**DOI:** 10.1186/s40902-018-0145-5

**Published:** 2018-03-12

**Authors:** Jung-Eun Park, Seon-Hye Bae, Young-Jun Choi, Won-Cheul Choi, Hye-Won Kim, Ui-Lyong Lee

**Affiliations:** 1 Department of Orthodontics, Dental Center, Chung-Ang University Hospital, Seoul, Korea; 2 Department of orthodontics, Estar dental clinic, Seoul, Republic of Korea; 3 Department of Oral & Maxillofacial Surgery, Dental Center, Chung-Ang University Hospital, Seoul, Republic of Korea

## Correction

The publication of this article [1] unfortunately contained several mistakes.

1. Typing errors occurred on page 1 (methods section in abstract), page 2 (background section and methods section), and page 6 (discussion section). The “prospective” should be read as “retrospective”.

In our institute, follow-up CT after surgery and collecting snoring data before and after surgery were routine procedures in all patients who receiving orthognathic surgery. This was a process to evaluate accurately the patient after the operation.

Therefore, the prospective data collecting has been done regardless of the approval of the ethics committee. However, IRB approval had been required for the analysis and publication for collected data. Consequently, IRB approval had been made for this retrospective study. The authors confused prospective data collecting and retrospective data analysis. The authors regret this confusion.

2. On page 3 the last sentence, “After surgery, the APL of the airway decreased by 2.3 mm and 1.6 mm in CV1 and CV2, respectively, and increased by 0.34 mm in CV3. The LTW of airway decreased by 1.9 mm, 2.3 mm and 0.7 mm in CV1, CV2, and CV3, respectively. Except the APL in CV3, all other APL and LTW measurements had a tendency to decrease, and the greatest changes were observed in APL of CV1 and LTW of CV2.” should be read as “After surgery, the LTW of the airway decreased by 2.3 mm and 1.6 mm in CV1 and CV2, respectively, and increased by 0.34 mm in CV3. The APL of airway decreased by 1.9 mm, 2.3 mm and 0.7 mm in CV1, CV2, and CV3, respectively. Except the LTW in CV3, all other APL and LTW measurements had a tendency to decrease, and the greatest changes were observed in LTW of CV1 and APL of CV2.”
3. On page 5 the second sentence on right column, “As for the ratio of change before and after the surgery in regards to measurements before surgery, the ratio of change in APL and CSA in CV1 showed significance in occurrence of snoring at 1% significance level” should be read as “As for the ratio of change before and after the surgery in regards to measurements before surgery, the ratio of change in LTW in CV1 showed significance in occurrence of snoring at 1% significance level”
4. An error occurred in Table 5 during the final stages of mounting.
  The corrected Table 5 is given below:

**Table 5:** The Correlation of the proportion of difference between before and after surgery to the values before surgery

	T1-T0/T1	Beta	SD	*P*-value
C 1 (Retropalatal)	LTW	−0.7602	0.2819	0.0070 ^***^
	APL	−0.1464	0.084	0.0813
	CSA	−0.2537	0.1047	0.0154 ^**^
C 2 (Retroglossal)	LTW	−0.0066	0.1285	0.9593
	APL	0.0222	0.0715	0.7557
	CSA	−0.0269	0.0483	0.5772
C 3 (Glottis)	LTW	0.1733	0.1382	0.2098
	APL	−0.1424	0.0729	0.0507 ^*^
	CSA	−0.0263	0.0535	0.6235

(^***^, ^**^, ^*^ represent 1%, 5%, 10% significance level respectively)

The authors apologize for those errors and state that this does not change the scientific conclusions.

This has now been included in this erratum.
